# Spectral Photon-Counting Molecular Imaging for Quantification of Monoclonal Antibody-Conjugated Gold Nanoparticles Targeted to Lymphoma and Breast Cancer: An *In Vitro* Study

**DOI:** 10.1155/2018/2136840

**Published:** 2018-12-18

**Authors:** Mahdieh Moghiseh, Chiara Lowe, John G. Lewis, Dhiraj Kumar, Anthony Butler, Nigel Anderson, Aamir Raja

**Affiliations:** ^1^Department of Radiology, University of Otago, Christchurch School of Medicine, 2 Riccarton Avenue, Christchurch 8011, New Zealand; ^2^Steroid & Immunobiochemistry Laboratory, Canterbury Health Laboratories, 524 Hagley Ave, Christchurch 8011, New Zealand; ^3^Department of Obstetrics and Gynecology, University of Otago, Christchurch School of Medicine, 2 Riccarton Avenue, Christchurch 8011, New Zealand

## Abstract

The purpose of the present study was to demonstrate an *in vitro* proof of principle that spectral photon-counting CT can measure gold-labelled specific antibodies targeted to specific cancer cells. A crossover study was performed with Raji lymphoma cancer cells and HER2-positive SKBR3 breast cancer cells using a MARS spectral CT scanner. Raji cells were incubated with monoclonal antibody-labelled gold, rituximab (specific antibody to Raji cells), and trastuzumab (as a control); HER2-positive SKBR3 breast cancer cells were incubated with monoclonal antibody-labelled gold, trastuzumab (specific antibody to HER2-positive cancer cells), and rituximab (as a control). The calibration vials with multiple concentrations of nonfunctionalised gold nanoparticles were used to calibrate spectral CT. Spectral imaging results showed that the Raji cells-rituximab-gold and HER2-positive cells-trastuzumab-gold had a quantifiable amount of gold, 5.97 mg and 0.78 mg, respectively. In contrast, both cell lines incubated with control antibody-labelled gold nanoparticles had less gold attached (1.22 mg and 0.15 mg, respectively). These results demonstrate the proof of principle that spectral molecular CT imaging can identify and quantify specific monoclonal antibody-labelled gold nanoparticles taken up by Raji cells and HER2-positive SKBR3 breast cancer cells. The present study reports the future potential of spectral molecular imaging in detecting tumour heterogeneity so that treatment can be tuned accordingly, leading to more effective personalised medicine.

## 1. Introduction

The current molecular imaging modalities, such as positron emission tomography (PET), single-photon emission-computed tomography (SPECT), magnetic resonance imaging (MRI), and optical coherence tomography (OCT), have come a long way towards the observation of biological processes at molecular and cellular levels. However, each of these modalities has its limitations that contribute to an inability in measuring specific biomarkers of cancer in patients [1–4]. PET and SPECT, although sensitive, are slow, nonspecific, and require radioactive tracers. MRI provides excellent soft tissue contrast but is slow, has poor spatial resolution, and cannot be used for patients with claustrophobia or metallic implants. OCT is sensitive and specific, but its limited penetration depth prevents it from being translated to most clinical tasks. To overcome the limitations associated with current biomedical imaging techniques, we aim to use new imaging technology at clinical X-ray energy ranges, with the inclusion of monoclonal antibody-functionalised gold nanoparticles.

The advent of the energy-discriminating photon-counting spectral detector [5] has opened the door to radically new approaches to medical investigation and monitoring. Like a prism splitting white light into a rainbow, a spectral detector captures full information in multiple X-ray energy bins. Spectral CT imaging combines the high-resolution anatomical detail of standard CT with the ability to characterise and quantify components of the tissue. As each material has a specific measurable X-ray spectrum, spectroscopic imaging can simultaneously measure several biomarkers of biological processes at the cellular and molecular level, using simultaneously acquired data for multiple energy bins. The combination of high spatial and spectral resolution with specific identification and quantification of multiple tissue components, noninvasively, is unique to specific cells and molecules. This cellular and molecular-specific CT imaging is known as *spectral molecular CT imaging.* As an emerging revolution in X-ray-based imaging, spectral molecular CT promises to complement the existing molecular imaging modalities and to be a potential tool for delivering personalised medicine [6–10]. Preclinical results with spectral molecular CT have been very encouraging and indicate that the specific identification and quantification of tissue types and nanoparticles is possible, such as imaging of vulnerable plaque [11, 12], soft tissue quantification [13], reduction in metal-related CT artefacts [14, 15], crystal-induced arthropathies [16, 17], quantitative imaging of excised osteoarthritic cartilage [18], and K-edge imaging of high-Z (atomic number) biomedical nanoparticles [19, 20].

Laboratory and preclinical results with spectral molecular CT have been very encouraging, but the question of how spectral molecular CT imaging could be used in clinical practice still remains. Given the evidence so far, spectral CT has the capability of determining drug delivery and host immune response to cancer, a clinical area that is not yet met by current clinical imaging modalities. In recent years, advances in nanotechnology and nanomedicine have focussed on targeting tumours for diagnosis and therapy [21–23]. Nanoparticles possess desirable physiochemical properties for chemical and biological detection, including improving signal strength in imaging, high surface area to volume ratio, and easily tuneable surface chemistry [23–25]. Nanoparticle-based drug delivery systems for systemic applications have significant advantages and have the potential to be more effective compared to their nonformulated, free drug counterparts, as surface chemistry allows the attachment of functional groups which recognise biological cues for improved specificity [26].

Among metal nanoparticles, engineered gold nanoparticles (AuNPs) are increasingly utilised in various biomedical applications due to their inert nature, low size dispersity (size distribution), high stability, comparably easy synthesis, noncytotoxic nature, and biocompatibility [27]. AuNPs have ideal properties for use as a targeted nanocontrast material for imaging cancer [28–35]. Spectral molecular CT integrated with nanoparticle technology may solve limitations faced by current molecular imaging modalities and facilitate drug discovery. The present study will employ AuNPs to label monoclonal antibodies for the reasons stated.

The aim of the present research is to test the ability of spectral molecular CT to detect and quantify the delivery of drugs to tumours, using targeted gold-labelled monoclonal antibodies. We are reporting for the first time MARS spectral photon-counting CT imaging of Herceptin-modified AuNPs, specific to HER2-positive breast cancer cells, as a way of establishing a novel multifunctional platform that allows for the identification of pathology and assessment of treatment. Moreover, rituximab, specific to Raji lymphoma cancer cells, will provide a crossover experiment. A negative control is an ideal scenario for a preclinical “proof of principle” showing the ability of MARS spectral CT imaging technology to distinguish and quantify specifically labelled cells.

## 2. Materials and Methods

### 2.1. Spectral Photon-Counting CT Imaging

#### 2.1.1. MARS Spectral CT Scanner Setup

MARS spectral CT is enabled by the properties of the photon-processing detector, Medipix3RX (Medipix3RX Collaboration, CERN), within the MARS spectral scanner [13]. Multiple energy windows or bins of the energy-resolving detector provide sufficient data to separate several materials in a single X-ray exposure [19]. The spectral CT technology has been reported to differentiate and quantify multiple different high-Z materials in a single scan by sampling the material-specific attenuation curves within multiple narrow energy bins, allowing the detection of element-specific K-edge discontinuities of the photoelectric cross section [36, 37].

Medipix3RX detector can be operated in either the single pixel mode (SPM) or charge summing mode (CSM) [38]. To cover the K-edge of Au, energy thresholds were set: 18, 30, 45, and 75 keV in CSM. Acquired data were reconstructed in four narrow CSM energy bins (18–30, 30–45, 45–75, and 75–118 keV) by a 3D algebraic reconstruction algorithm [39] (Table 1).

#### 2.1.2. Image Processing

Prior to scanning the samples, the MARS system creates a pixel mask by acquiring 20 dark-field (without X-rays) and 200 flat-field (open beam) images. This mask was applied to remove noisy pixels, including nonfunctional and high and low sensitivity pixels [37, 40, 41]. For each study, the calibration vials (2, 4, and 8 mg/mL AuCl_3_·*x*H_2_O) were placed within a phantom holder, along with the cell vials, and scanned. The assessment of the reconstructed images involved measuring the linear attenuation for each material and converting linear attenuation into Hounsfield units (HU). Using an in-house programme that uses the calculated effective mass attenuation of the calibration vials [20, 39], material decomposition (MD) was applied to the energy images and quantification of the trastuzumab and rituximab was performed by measuring the amount of gold in the cell clumps.

#### 2.1.3. Cancer Cell Phantoms

200 *µ*L Eppendorf tubes of each material (Table 2) were placed in a polymethyl methacrylate (PMMA) phantom holder. One vial with SKBR3 was incubated with trastuzumab-gold nanoparticle complex (Her-AuNP). As a control, SKBR3 incubated with rituximab gold nanoparticle complex (Rit-AuNP) was included. Raji lymphoma cells were treated with the same protocol, incubated with Her-AuNP and Rit-AuNP, in a separate PMMA phantom holder. Water, lipid, and 3 concentrations of AuNPs (2, 4, and 8 mg/mL) were used for calibration purposes in both phantoms.

### 2.2. Gold Nanoparticles

The streptavidin-modified gold nanoparticles of size 40 nm were purchased from Fitzgerald Industries, Acton, MA, and characterised by the UV-visible spectrophotometer and dynamic light scattering (Malvern Nano Zetasizer ZS) to confirm the material, size, and size distribution.

The absorption spectrum of gold nanoparticles caused by surface plasmon absorption is directly related to the nanoparticle size. Surface plasmon resonance (SPR) is the coherent excitation of all free electrons within the conduction band. As particle size increases, the wavelength of SPR related to absorption shifts to longer, redder wavelengths [42, 43]. SPR is important as it allows the AuNP size to be specifically identified so that it is the gold added to the experiment which is being measured.

### 2.3. Antibodies and Labelling

#### 2.3.1. Antibodies

Trastuzumab (Herceptin, Roche Pharmaceuticals, Mississauga, ON, Canada) binds exclusively to HER2-positive human breast cancer cells, and rituximab (Mabthera) binds to CD20 antigen on human B-cell lymphomas and are humanised chimeric therapeutic monoclonal antibodies with a human Fc domain. Both drugs were made and supplied by Roche Pharmaceuticals, Mississauga, ON, Canada. Goat antihuman IgGFc-biotin was from Fitzgerald Industries, Acton, MA, and streptavidin peroxidase was supplied by Jackson Immunoresearch Laboratories, West Grove, PA.

#### 2.3.2. Biotinylation of Antibodies

Rituximab and trastuzumab were both dialysed, and then each were diluted to 2.5 mg/mL with 0.1 M borate buffer, pH 8.8, 1/20th volume of biotinamidocaproate N hydroxysuccinamide ester in DMSO (10 mg/mL) added (goat antihuman IgGFc-biotin, Fitzgerald Industries, Acton, MA) and then mixed for four hours at 200°C. Unreacted biotin ester was blocked by the addition of 1 M ammonium chloride. Following exhaustive dialysis against phosphate-buffered saline (PBS), the material was clarified by centrifugation and concentration adjusted to 2 mg/mL.

### 2.4. *In Vitro* Studies

#### 2.4.1. Cell Lines

The HER2-positive human breast cancer line SKBR3 and the CD20-positive human B-cell line Raji cells were obtained from frozen stocks held by the Steroid and Immunobiochemistry Laboratory, Canterbury Health Laboratories, Christchurch, New Zealand. They were cultured in flasks in RPMI 1640 supplemented with 10% foetal calf serum (FCS) media and grown at 37°C in 5% CO_2_. The SKBR3 cells were harvested at confluency and the Raji cells during log phase growth. Following harvest, the cells were centrifuged and washed twice with PBS. The cells were then used for cell-based ELISA experiments to optimise incubation and labelling conditions for subsequent labelling with antibody-AuNPs and analysis by spectral CT scanning. The use of both SKBR3 and Raji cells allowed crossover control experiments for each monoclonal antibody as SKBR3 HER2 positive but CD20 negative; and Raji cells are CD20 positive but HER2 negative.

#### 2.4.2. Cell-Based Enzyme-Linked Immunosorbent Assay (ELISA)

Washed cells were suspended into 11 mL of PBS and plated across the wells (100 *µ*L/well containing 20,000 cells) of a 96-well, flat-bottomed, microtiter plate. The plate was centrifuged for 5 minutes to sediment the cells, and the PBS was carefully aspirated. Cells were fixed for 30 minutes at 20°C by adding 2% paraformaldehyde into the PBS (100 *µ*L/well). The paraformaldehyde was then carefully aspirated, and the plate was dried under a gentle stream of air. The plate was then blocked with assay buffer containing 1% FCS for 30 minutes at 20°C (200 *µ*L/well). Following blocking, the buffer was decanted, and the plate was blot dried by inversion. 2 mg/mL dilutions of either rituximab, trastuzumab, biotinylated rituximab, or biotinylated trastuzumab were added for 30 minutes at 20°C. The wells were then washed three times (200 *µ*L/well). For the rituximab and trastuzumab series, antihuman IgGFc-biotin was added for another 30 minutes at 20°C (1 : 1000 in PBS containing 1% FCS and 100 *µ*L/well). For the biotinylated rituximab and biotinylated trastuzumab series, the plates were washed and 100 *µ*L/well of 1 : 1000 dilution of streptavidin peroxidase (Fitzgerald Industries, Acton, MA) was added for 30 minutes at 20°C, followed by washing and addition of tetramethylbenzidine substrate (100 *µ*L/well). Following the addition of antihuman IgGFc-biotin rituximab and Herceptin series, the plates were washed and incubated with 1:1000 streptavidin peroxidase for 30 minutes at 20°C prior to the final washing and the addition of tetramethylbenzidine substrate (100 *µ*L/well). Following colour development, the reaction was stopped by the addition of 1 M HCl (100 *µ*L/well), and the absorbance was read at 450 nm. The absorption reading gives an indication of the particle size.

#### 2.4.3. Gold Labelling of Cells

SKBR3 (20–100 million) and Raji (20–100 million) cells were harvested, washed as described, and each cell type was divided into two portions. Each portion was incubated with either Herceptin or rituximab (1 : 100 of 2 mg/mL in PBS containing 1% FCS) for 30 minutes at 20°C in 1.5 mL Eppendorf tubes and continually mixed. The cells were then washed and antihuman IgGFc-biotin (1 : 100 in PBS containing 1% FCS) was added for 30 minutes at 20°C. Following three washes in PBS, streptavidin-Au was added (1 : 10 in PBS containing 1% FCS) for a further 30 minutes at 20°C. The cells were finally washed three times in PBS and transferred to 0.5 mL Eppendorf tubes and pelleted for MARS scanning.

## 3. Results

### 3.1. Gold Nanoparticle Characterisation

The streptavidin-modified AuNPs of size 40 nm showed the lambda max peak at 530 nm. Similar observations have been reported in literature, concluding the expected size as 40 nm [44]. The dynamic light scattering (DLS) confirmed the NPs hydrodynamic size to be 50 nm (expected 40 nm). The standard absorbance graph obtained has been included in supporting information along with a typical size distribution graph by dynamic light scattering (Figure S1).

**Figure 1:**
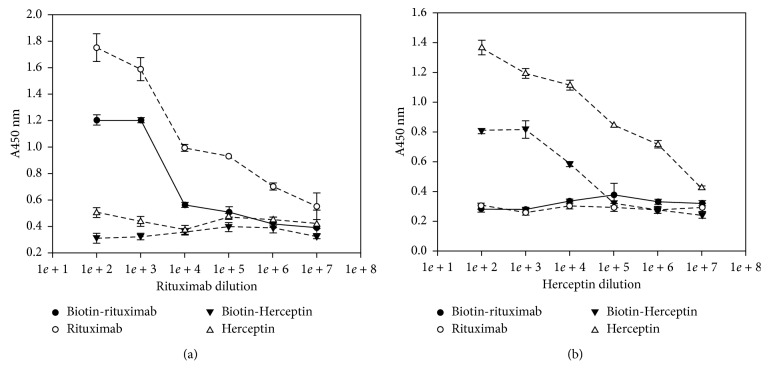
The quantitative data collected using the ELISA kit in the form of absorbance against drug dilution. The absorbance was read at 450 nm for (a) rituximab dilution in the case of Raji cells and (b) Herceptin dilution in the case of SKBR3.

The cell-based ELISA studies on the immobilised CD20-positive Raji cell line showed higher responses with rituximab dilutions and antihuman IgG-biotin and streptavidin peroxidase compared with the same dilutions of biotinylated-rituximab and streptavidin peroxidase (open and filled circles, respectively,, in Figure 1(a)). Similarly, cell-based ELISA studies on immobilised confluent HER2-positive human breast cancer cells (SKBR3) showed higher responses with the Herceptin, antihuman IgGFc-biotin, and streptavidin peroxidase combination compared with the biotinylated-Herceptin and streptavidin peroxidase combination (open and closed triangles, respectively, in Figure 1(b)). The trastuzumab and rituximab controls are also shown in Figures 1(a) and 1(b) and show that the two antibodies can clearly be distinguished in a crossover study.

Hence, for the present study, the former combination, with the addition of streptavidin-Au in the final step, was chosen with the rationale that this combination would provide a higher gold payload for scanning. Indeed, we confirmed this rationale by small-scale experiments and visualisation of cell-bound gold nanoparticles (data not shown).

### 3.2. Spectral Photon-Counting CT Imaging

**Figure 2:**
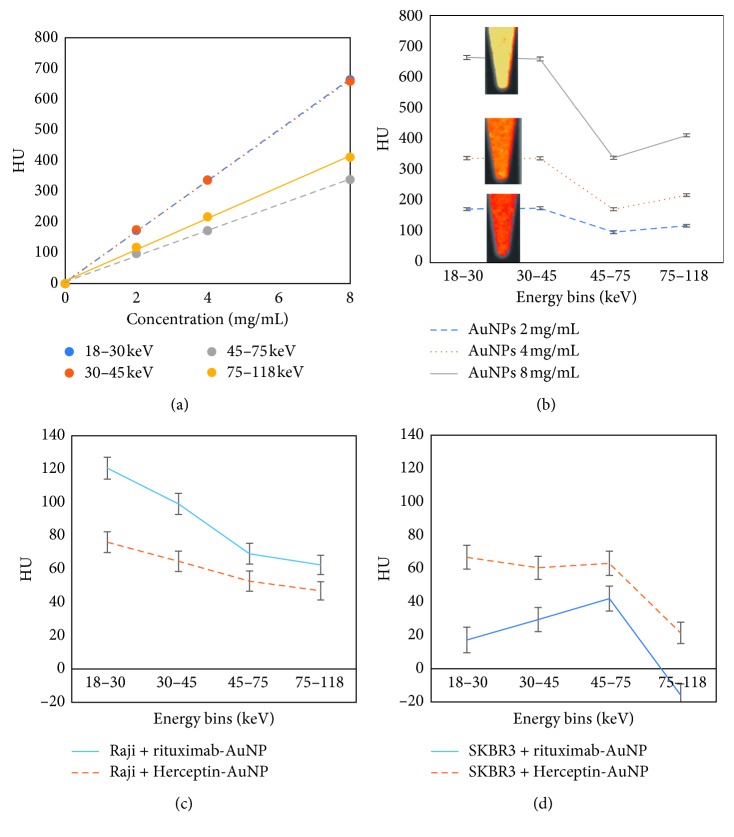
(a) Linearity of attenuation of calibration vials containing AuNP for each energy bin. *R* ^2^ = 0.99 for all linearity trends. (b) Spectral response of the detector for calibration vials. Enhancement of attenuation due to K-edge observed in energy bin 4 (75–118 keV). Insets show material decomposed images corresponding to each concentration. (c) and (d) Spectral response of Raji and SKBR3 cells, respectively. Standard errors are shown for spectral response data.

The attenuation signal in Hounsfield units (HU) as a function of concentration data has been included in Figure 2(a). By fitting a line that best describes the data, the linearity of attenuation for each energy bin was established [45]. Linearity determines the ability of a system to detect the presence or absence of any materials. This information directly feeds into the material quantification. Furthermore, the spectral response of the detector was plotted in Figure 2(b). Considering the K-edge of gold to be 80.7 keV, and an energy threshold set at 75 keV, we observed, as expected, an enhancement of attenuation in energy bin 4 (75–118 keV) for each concentration of AuNPs.

**Figure 3:**
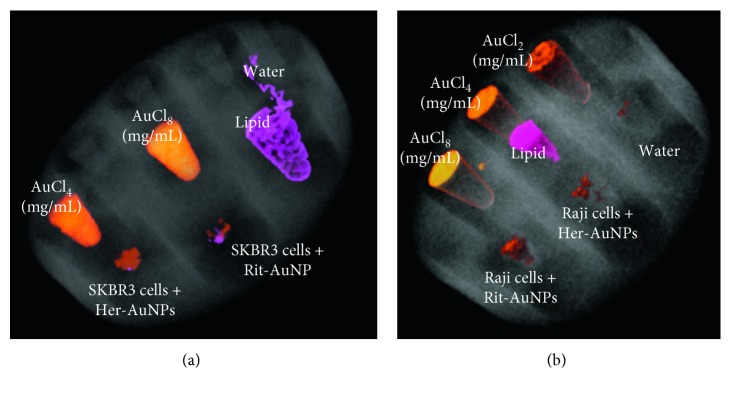
(a) SKBR3 cell line phantom. SKBR3 cells with Herceptin-AuNPs show more volume, indicating more gold is present. Material decomposition basis images show Au (yellow/orange, the hue represents the concentration), lipids (pink), and water-like material (grey). (b) Raji cell line phantom; Raji cells with rituximab-AuNPs show more volume and brighter hue, indicating a high concentration of gold present.

**Figure 4:**
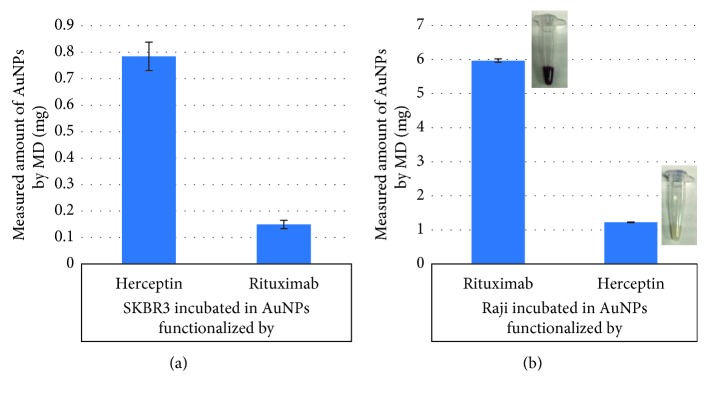
(a) Measured amount of targeted and nontargeted AuNPs (mg) within SKBR3 quantified from MD images displayed. (b) Measured amount of targeted and nontargeted AuNPs (mg) within Raji cells calculated from MD images. Insets show Raji cells plus Rit-AuNPs and Raji cells plus Her-AuNPs.

Greater HU was observed for Raji cells with rituximab-functionalised AuNPs and SKBR3 with Herceptin-functionalised AuNPs, as shown in Figures 2(c) and 2(d), respectively. By only observing the spectral response of the detector, spectral CT imaging is capable of indicating the presence of an attenuating material: the gold attached to trastuzumab, thus showing trastuzumab uptake into the SKBR3 cells and rituximab uptake into Raji cells. Furthermore, Figures 3(a) and 3(b) visualised, using MARS material decomposition, the detection of AuNPs in SKBR3 cells and Raji cells, respectively. Quantification of the resulting MD images was performed and shown in Figures 4(a) and 4(b). A significantly lower amount of AuNPs was detected and quantified in the control cells. Eightfold lower in SKBR3 cells incubated with rituximab modified AuNPs, and sixfold lower in Raji cells incubated with Herceptin modified AuNPs.

## 4. Discussion

The key outcomes of this *in vitro* study are first that spectral photon-counting CT can measure gold-labelled specific antibodies targeted to specific cancer cells; and second, that NPs can be integrated with spectral CT to generate a combined diagnostic imaging and a therapeutic agent, which can be detected and monitored. The concepts of using AuNPs with biotin to deliver a drug are well known [46]. Rituximab has been delivered using AuNPs [47]. Herceptin delivered by indium radiolabelled AuNPs has been shown to be cytotoxic *in vitro* [48]. Methods of using biotin and streptavidin with antibodies to target HER2-positive tumours are an established way [49]. In this study, we utilised these established methodologies and demonstrated the proof of principle that spectral molecular CT imaging can identify and quantify specific monoclonal antibody-labelled gold nanoparticles taken up by Raji cells and HER2-positive SKBR3 breast cancer cells.

Medical imaging is key to the diagnosis or assessment of disease response in many areas of medicine. Nevertheless, measuring disease activity, host response, and the effectiveness of treatment is frequently indirect, slow, and qualitative unless invasive procedures are performed. SKBR3, a breast cancer cell line which overexpresses epidermal growth factor receptor 2 (HER2), and Herceptin monoclonal antibody were used to test both the molecular specific binding of functionalised AuNPs and MARS spectral CT imaging. Herceptin works by attaching to HER2 on the cancer cells and inhibiting intracellular signalling. Crossover experiment was conducted to further support our findings. Rituximab, a monoclonal antibody which destroys both normal and malignant B cells that have CD20 receptors on their surface, is used to treat diseases that have overexpressed or dysfunctional B cells, for instance, Raji cells. Using multiple energy thresholds, the broad X-ray spectrum was divided into narrow energy bins to discriminate gold, water, and lipid. Establishing linearity of attenuation in each energy bin essentially creates multiple monochromatic CTs from a polychromatic CT X-ray source [45]. Accurate linearity of the system for any material (*R* ^2^ value ≈ 0.99 for all four energy bins was established) validates the quantification of that material. During the course of data analysis, we observed hidden K-edge phenomenon [50], which could be associated with low concentrations of AuNPs uptake by Raji and SKBR3; no enhancement of attenuation in energy bin 4 (Figures 2(c) and 2(d)) [50, 51]. However, our material decomposition algorithm [50] was able to recover this hidden information using the effective linear attenuation for each material (for each concentration and energy bin), which was estimated by taking the mean of respective regions in the reconstructed data, and demonstrates the uniqueness of spectral CT imaging [20].

Anticancer drugs are often hydrophobic, and attaching nanoparticles is a way of transporting the toxic drugs safely to the target site, where the drug will then be taken up by the cell and become active [52]. Molecules are too small to image directly; therefore, specific and sensitive contrast material is used to overcome this limitation and enhance targeted pathology. Nanoparticles provide unique and desirable physiochemical properties, ideal for CT imaging. A study by Kumar et al. suggested that size and cell type influenced the uptake of “as prepared” AuNPs by ovarian cancer cells [53]. Optimisation of the cellular uptake of AuNPs is required for different cell types to achieve enough payload. Functionalisation of AuNPs, or active uptake, improves specificity and payload. Examining molecular abnormalities of disease via noninvasive molecular imaging has allowed earlier detection, disease progression monitoring, and treatment assessment. This is achieved as molecular imaging plays a primary role in the optimisation of pathology, locating pathological lesions, guiding surgery and biopsy, and enabling more accurate diagnostic decision-making by the oncologist. The target-specific molecular probe is a crucial aspect for the development of diagnostic and therapeutic methods that address personalised treatment. Treatment focus becomes the individual patient rather than the disease [54]. Herceptin is often used with other chemotherapy medication for the treatment of breast cancer. Studies have shown slower tumour growth, which has a profound effect on the course of disease and survival of women with aggressive HER2-positive breast cancer [55–58]. Spectral imaging is a new frontier in molecular imaging which, if, used in conjunction with nanoparticle contrast material has the potential to accelerate the study of pharmacokinetics and the development of drugs for the treatment of cancer.

Distinguishing HER2-positive breast cancer cells from other cancer cell lines with photon-counting spectral CT will allow tumour heterogeneity to be imaged, spatially located, and measured noninvasively. ER expression in breast tumours could be targeted at the same time in future studies. It is hypothesized that the current nonspecific imaging approach where most cancers are staged with imaging, then reimaged to assess tumour size to see if treatment is working, or with PET to see if the metabolic activity has reduced, implying treatment is working, will be transformed. Moreover, current pathological methods to assess biopsy and whole breast specimens are unable to determine tumour heterogeneity mostly because specimen has to be cut into sections and sampled. Heterogeneity can be underreported or missed due to sampling errors. These results may also pave the way towards providing a new approach to characterise breast cancer cell types within the specimens, where whole biopsy specimens can be used with multiple targeted markers in conjunction with spectral CT to map out and specifically identify the different populations of breast cancer cells (or different cancer cells in case of different tumour types) within the specimen, identifying and measuring breast cancer heterogeneity.

Our results illustrate the proof of concept that spectral molecular CT imaging in conjunction with functionalised gold nanoparticles can provide valuable information on tumour heterogeneity while providing important physiologic data at the cellular level. The proof of principle reported in the current study has the potential to have a major impact on the management of cancers that express specific biomarkers, such as the expression of HER2. With regard to other potential models, HER2 has also been suggested as a target for lung [59] and colorectal cancers [60]. This laboratory methodology is specifically designed to translate to human imaging so that in the future, women with breast cancer can have their treatment tuned to match changes in their tumour. The significance of this work is that once spectral CT scanners are available for clinical use, clinicians will in future be able to monitor HER2 receptor status of all sites of breast cancer in an individual, detect how and where this status changes, monitor drug delivery and disease response, and adjust treatment to keep pace with these biological changes in breast cancer, that is, be able to detect and respond to tumour heterogeneity. In this context, it is important to note that spectral CT has recently progressed to imaging the first ever human scan [61].

## 5. Conclusion

AuNPs are being studied and developed as a promising multifunctional platform for imaging and drug delivery applications. The present study successfully established *in vitro* methodology that aimed to measure gold-labelled specific monoclonal antibodies targeted to specific cancer cells; trastuzumab and rituximab to HER2-positive SKBR3 breast cancer and Raji B-cell lymphoma, respectively. Spectral photon-counting CT results show that Raji cells and HER2-positive SKBR3 breast cancer cells take up gold nanoparticles, if the nanoparticles are conjugated with a monoclonal antibody specific to them. A key issue that was observed and fulfilled during the study was to load the optimal amount of AuNPs for quantitative and qualitative analyses using spectral CT. The information provided is relevant for the development of more sensitive and specific targeted imaging tests for various malignancies using a range of off-the-shelf, stable functionalised nanoparticles. Spectral imaging has the potential to allow accurate diagnosis of tumour type, size, and location. The methodology in the present study was designed to translate to human imaging so that in the future, tumour heterogeneity can be detected, and treatment tuned accordingly, leading to more effective personalised cancer treatment.

## Figures and Tables

**Table 1:** Summary of MARS spectral CT scanner experimental setup.

Parameter	Value
Scan type	Continuous
Tube voltage	118 kVp
Tube current	12 *µ*A
Exposure time	300 ms
Sample diameter	38 mm
Energy (CSM)	18, 30, 45, and 75 keV
Circular projections	720 over 360°
Flat fields	720
Voxel size	1 × 1 × 1 mm^3^
SDD, SOD	250 mm, 200 mm
Filtration	2 mm Al + 1.8 mm Al intrinsic

SDD: source-to-detector distance; SOD: source-to-object distance.

**Table 2:** Summary of phantom setup.

Raji phantom	SKBR3 phantom
AuNPs 2, 4, and 8 mg/mL	AuNPs 2, 4, and 8 mg/mL
AuNPs size by DLS: 50 nm (expected 40 nm)	
AuNPs absorbance: 530 nm	
Water, lipid	Water, lipid
Raji cells with Her-AuNP (control)	SKBR3 with Her-AuNP
Raji cells with Rit-AuNP	SKBR3 with Rit-AuNP (control)

Her, trastuzumab or Herceptin; Rit, rituximab.

## Data Availability

The data used to support the findings of this study are available from the corresponding author upon request.

## Supplementary Materials

Supplementary Materials Figure S1: gold nanoparticle characterisation using ultraviolet-visible spectra and a maximum absorption at 530 nm, observed in literature. Figure S2: extended methods for the quantification of material-decomposed (MD) results from MARS computed tomography molecular imaging, indicating the reliability and accuracy of MD results.
