# Supplementary material for: Spectral Photon-Counting Molecular Imaging for Quantification of Monoclonal Antibody-Conjugated Gold Nanoparticles Targeted to Lymphoma and Breast Cancer: An In Vitro Study
Source: Contrast Media Mol Imaging. 2018 Dec 18;2018:2136840. doi: 10.1155/2018/2136840 (PMC6312585; doi:10.1155/2018/2136840)
Supplement: Supplementary Materials — Figure S1: gold nanoparticle characterisation using ultraviolet-visible spectra and a maximum absorption at 530 nm, observed in literature. Figure S2: extended methods for the quantification of material-decomposed (MD) results from MARS computed tomography molecular imaging, indicating the reliability and accuracy of MD results. [file 2136840.f1.pdf]

Supplementary Materials

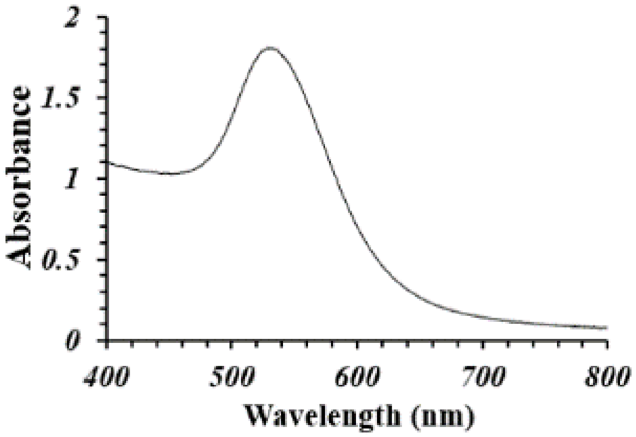

Figure S1: UV-Visible spectra of 40 nm AuNPs. Peak in absorption observed at 530 nm.

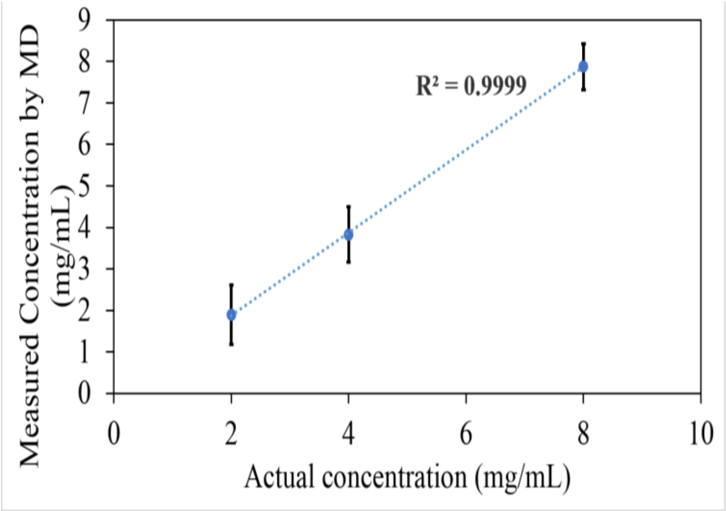

Figure S2: Quantification of AuNP calibration vials. Measured concentration from MD images versus actual concentration for known concentration of AuNPs.
